# Effect of SARS-CoV-2 Infection and COVID-19 Vaccination on Oxidative Status of Human Placenta: A Preliminary Study

**DOI:** 10.3390/antiox12071403

**Published:** 2023-07-09

**Authors:** Kristína Macáková, Petra Pšenková, Nadja Šupčíková, Barbora Vlková, Peter Celec, Jozef Záhumenský

**Affiliations:** 1 Institute of Molecular Biomedicine, Faculty of Medicine, Comenius University, 81108 Bratislava, Slovakia; kristina.macakova@imbm.sk (K.M.); nadja.ivaskova@imbm.sk (N.Š.); barbora.vlkova@imbm.sk (B.V.); 2 2nd Department of Gynaecology and Obstetrics, University Hospital Bratislava and Comenius University, 82606 Bratislava, Slovakia; petrapsenkova@gmail.com (P.P.); jozef.zahumensky@ru.unb.sk (J.Z.); 3 Institute of Pathophysiology, Faculty of Medicine, Comenius University, 81108 Bratislava, Slovakia

**Keywords:** virus, inflammation, oxidative damage, cell-free DNA, pregnancy

## Abstract

Infection with SARS-CoV-2 during pregnancy increases the risk of pregnancy complications associated with inflammation, which could lead to oxidative stress in the placenta. Whether vaccination against COVID-19 has any effect is unclear. This study aimed to analyze the effects of SARS-CoV-2 infection and vaccination against COVID-19 during pregnancy on oxidative stress in the placenta and on extracellular DNA (ecDNA) in umbilical cord plasma. Placenta samples from healthy uninfected and unvaccinated control patients who recovered from COVID-19 and women vaccinated against COVID-19 during pregnancy were collected. Biomarkers of oxidative damage and antioxidant capacity were assessed in the placenta homogenates. EcDNA and deoxyribonuclease activity were quantified in umbilical cord plasma using real-time PCR and the single radial enzyme diffusion method, respectively. Markers of oxidative damage to lipids and proteins as well as antioxidant capacity in the placenta did not differ between the study groups. No differences were observed in total, nuclear or mitochondrial ecDNA, or deoxyribonuclease activity in the umbilical cord plasma. Taking into account the limits of a small observational study, our results suggest that the infection with SARS-CoV-2 and vaccination against COVID-19 do not induce any major disturbances in the balance between the production of free radicals and antioxidant activity in the placenta. This is in line with the minor effects on fetal outcomes and ecDNA as a suggested marker of fetal well-being.

## 1. Introduction

SARS-CoV-2, severe acute respiratory syndrome coronavirus 2, is a respiratory pathogen causing COVID-19 [1]. SARS-CoV-2 infection is associated with increased cytokine production [2] which creates a pro-inflammatory environment, resulting in tissue damage in the lung and elsewhere [3]. Inflammation caused by distinct pathways, either directly by the virus or indirectly through tissue damage, can lead to a disturbance in oxidative status. The subsequent increased formation of reactive oxygen species can lead to blood clotting and, eventually, organ failure [4,5]. Pregnancy is a risk factor for respiratory and other infections due to transient changes in the immune system [6]. Oxidative stress is increased even during a physiological pregnancy due to high metabolic demands to ensure adequate fetal development [7].

Oxidative balance plays an essential role in the regulation of placental physiology [8], and disturbances in this balance can result in adverse consequences such as preeclampsia, gestational diabetes, or placental insufficiency [9]. Meta-analyses have shown that SARS-CoV-2 infection during pregnancy is associated with an increased risk of maternal mortality and preterm birth [10,11,12,13]. Inflammation of the placenta also belongs to the pathogenesis of pregnancy complications during infection [14]. The so-called SARS-CoV-2 placentitis is characterized by trophoblast necrosis and perivillous fibrin deposition [15,16]. In addition to the mentioned pathological changes, SARS-CoV-2 infection can result in the modulation of the microbiota, leading to gastrointestinal symptoms, but infection may also trigger the formation and the release of toxin-like peptides, resulting in the stimulation of the gut–brain axis [17,18]. Vaccination against COVID-19 can reduce severe consequences of SARS-CoV-2 infection. Studies analyzing the effects of COVID-19 vaccination during pregnancy do not indicate any increased risk of pathological changes in the placenta [19,20]. Placental oxidation as part of apoptosis or necrosis leads to increased release of extracellular DNA (ecDNA) [21]. EcDNA can thus be applied as a marker of tissue damage during SARS-CoV-2 infection or after vaccination against COVID-19 [22]. EcDNA is also present in the circulation of healthy individuals and its turnover is mainly controlled by cleavage enzymes—deoxyribonucleases (DNases) [23]. Insufficient removal of ecDNA can lead to inflammation, oxidative stress, and increased ecDNA [24]. Our study aimed to describe the effect of SARS-CoV-2 infection and vaccination against COVID-19 on markers of oxidative damage and antioxidant status in the placenta as well as ecDNA and DNase activity in umbilical cord plasma as a marker of fetal tissue damage.

## 2. Materials and Methods

### 2.1. Population/Subjects and Sampling of Placenta

**Figure 1:**
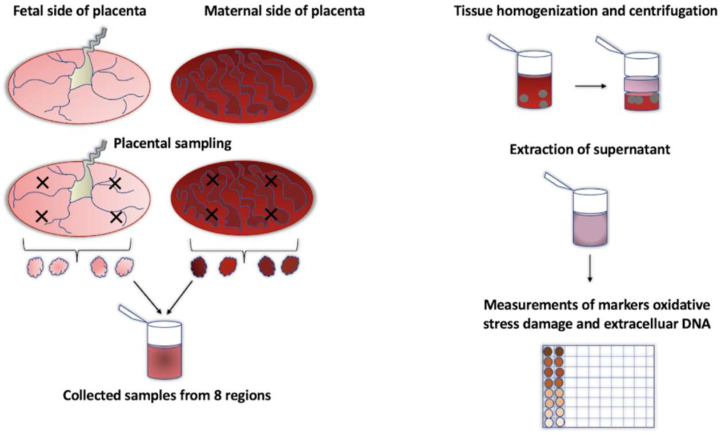
Processing of placenta samples.

This study involved 126 women who gave birth at the 2nd Department of Gynaecology and Obstetrics Clinic of the University Hospital Bratislava in Slovakia during July and August 2021. Clinical samples were collected based on the approval and signing of the informed consent. The clinical history of the women included high blood pressure and varices, disorders of the thyroid gland, endometriosis, polycystic ovary syndrome, and cervix dysplasia. Autoimmune diseases, atopic eczema, celiac disease, allergies, and cholecystitis were present in some of the participants. Participating women did not differ between the groups in the prevalence of comorbidities. Human placenta and umbilical cord plasma samples were collected from three groups of women (controls: n = 83, recovered from COVID-19: n = 23, vaccinated against COVID-19—all mRNA vaccines: n = 20) and immediately stored at −20 °C until further processing. Tissue samples were prepared by collecting placental tissue from eight regions, four from the maternal side and four from the fetal side, as previously described [25] (Figure 1).

The process of preparing tissue samples involved the collection of placental tissue from eight specific regions, with four regions taken from the maternal side and four from the fetal side. The collected samples were homogenized with the aim of obtaining a supernatant, which was further used in downstream processes.

### 2.2. Homogenization

Pooled tissue samples (100 mg) were homogenized in 1 mL of phosphate-buffered saline. Samples were homogenized at 20 kHz for 2 × 1 min using stainless steel beads in Qiagen TissueLyser II Bead Mill (Qiagen, Hilden, Germany). After homogenization, the tubes were centrifuged at 3000× *g* for 5 min. The supernatant was used for further analyses.

### 2.3. Biochemical Analysis of Oxidative Stress Markers

Oxidative damage was assessed using established biomarkers such as thiobarbituric acid reactive substances (TBARS), advanced oxidation protein products (AOPP), advanced glycation products (AGEs) and fructosamine. TBARS, as biomarkers of lipid peroxidation, were measured as previously described [26]. The calibration curve was prepared using 1,1,3,3-tetramethoxypropane. The final fluorescence was measured at λex = 515 nm and λem = 553 nm. For AOPP measurement, samples were mixed with glacial acetic acid for 2 min. As a standard, chloramine T and potassium iodine were used. Absorbance was measured at 340 nm [27]. AGEs were used as markers of carbonyl stress and quantified according to the published protocol [28]. AGE-modified bovine serum albumin was used as a standard. AGEs were determined spectrophotometrically at λex = 370 nm, λem = 440 nm. For fructosamine measurement, 16 mmol/L 1-deoxy-morpholino-D-fructose was used as a standard. Homogenates from placenta samples and standards were mixed with nitro blue tetrazolium containing 1 mmol/L nitro blue tetrazolium and 0.1 mol/L sodium carbonate buffer (pH = 10.35). Prepared samples were incubated at 37 °C for 15 min. Absorbance from the samples and standards was measured at 530 nm [29].

To characterize the antioxidant status, total antioxidant capacity (TAC) and ferric-reducing antioxidant power assay (FRAP) were used. Samples were mixed with the acetate buffer (pH = 5.8) and the initial absorbance at 660 nm was measured. After measuring the blank, 2,2′-Azino-bis-(3-ethylbenzothiazoline-6-sulfonic acid) with acetate buffer was added. Absorbance was measured again at 660 nm. The final TAC was calculated from the difference between the two measured absorbances [30]. For FRAP quantification, placenta homogenates were mixed with tripyridyl-s-triazine FeCl_3_. For standard, 100 mmol/L of FeSO_4_·7H_2_O was used. Absorbance was measured at 530 nm [31].

All assessed biomarkers were normalized to total protein concentration. To quantify total proteins in the samples, a bicinchoninic acid kit was used according to the protocol of the manufacturer. For the calibration curve, we used the bovine serum albumin standard set (Fermantas, Vilnius, Lithuania). If not otherwise stated, all used chemicals were purchased from Sigma Aldrich (Darmstadt, Germany).

### 2.4. DNA Isolation and Quantification

The ecDNA was isolated from the EDTA umbilical cord plasma using a QIAamp DNA Blood Mini Kit (Qiagen, Hilden, Germany) and a QIAcube device (Qiagen, Hilden, Germany). For the plasma separation, blood was centrifuged at 1600× *g* for 10 min at 4 °C. Obtained plasma was centrifuged again at 16,000× *g* for 10 min at 4 °C [32]. Quantification of ecDNA was performed using a Qubit 3.0 fluorometer and Qubit dsDNA high sensitivity assay (Thermo Fisher Scientific, Waltham, MA, USA). Subcellular origin of ecDNA was determined using quantitative PCR recorded with real-time PCR Mastercycler realplex 4 (Eppendorf, Hamburg, Germany). PCR mixture consisted of SYBR Green Supermix (Bio-Rad Laboratories, Hercules, CA, USA) and commercially prepared primers (Microsynth, Balgach, Switzerland) encoding part of human β-globin gene (ncDNA: F:5′-TGTCAGATATGTCCTTCAGCAAGG-3′, R:5′-TGCTTAACTCTGCAGGCGTATG-3′) and D-loop of the human mitochondrial gene (mtDNA: F:5′-CCCAGCTACTACCATCATTCAAGT-3′, R:5′-GATGGTTTGGGAGATTGGTTGATGT-3′). The PCR program consisted of one cycle of 98 °C for 3 min, 40 cycles of 98 °C for 15 s, 60 °C for 30 s, and 72 °C for 30 s.

### 2.5. DNase Activity

To determine DNase activity in umbilical cord plasma, 5 µL of samples was used in a single radial enzyme diffusion (SRED) assay. SRED was performed in a 1% agarose gel (20 mM Tris–HCl, pH 7.5, 2 mM MgCl_2_, 2 mM CaCl_2_) with DNA isolated from chicken livers (0,035 mg/mL per gel). To visualize DNA in the gel after digestion, GoodView Nucleic Acid Stain was used (SBS Genetech, Beijing, China). As a calibration, 1 µL of DNase and RDD buffer was applied (Qiagen, Hilden, Germany). After overnight incubation at 37 °C in the dark, the gel was photographed with iBOX (Vision works LP Analysis Software, UVP, Upland, CA, USA). Formed radial DNase diffusion in the gel as a result of DNase activity was then calculated using ImageJ software (NIH, Bethesda, MD, USA). The DNase activity was expressed in Kunitz units (KU) per mL (KU/mL) of umbilical cord plasma [33].

### 2.6. Statistical Analysis

Data obtained in this study were analyzed using the software GraphPad Prism 8.1 (La Jolla, CA, USA). The normality of data distribution was tested with the test for normality of distribution. Differences were analyzed using one-way parametric and non-parametric ANOVA. Data are shown as mean and standard deviation. Significant differences were considered when *p* < 0.05.

## 3. Results

**Figure 2:**
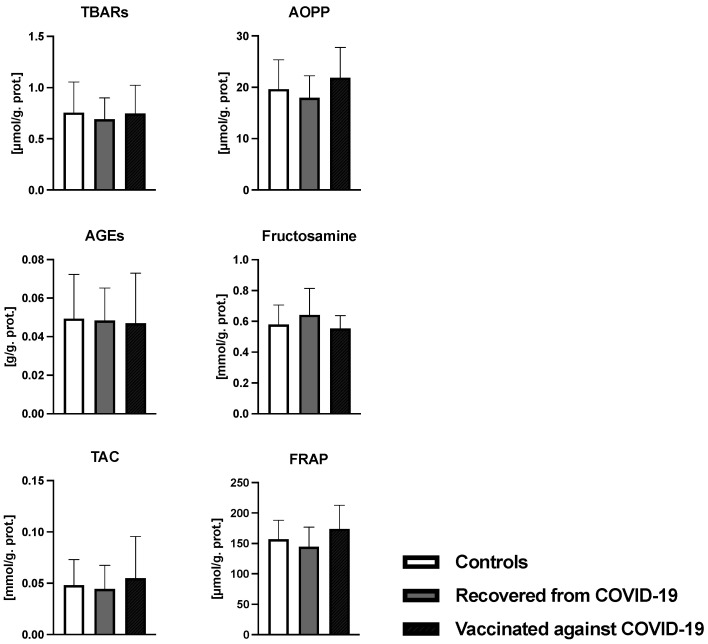
Concentration of thiobarbituric acid-reacting substances (TBARS), advanced oxidation protein products (AOPP), advanced glycation end products (AGEs), fructosamine, total antioxidant capacity (TAC), ferric-reducing antioxidant power (FRAP), and ratio (GSH/GSSG) of the oxidized and reduced glutathione. Differences between the selected groups were assessed with the ANOVA test. Data are shown as mean and standard deviation.

**Figure 3:**
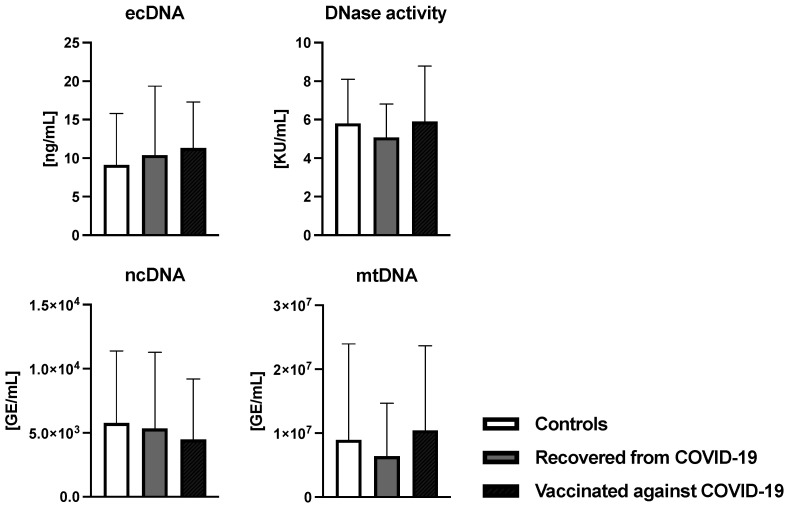
Concentration of total extracellular DNA (ecDNA), nuclear DNA (ncDNA), and mitochondrial DNA (mtDNA) in umbilical cord plasma. Differences between the selected groups were assessed with the ANOVA test. Data are shown as mean and standard deviation.

Table 1 summarizes the clinical parameters of the women who participated in the study. Of the total 126 women, 23 women recovered from COVID-19, 20 women were vaccinated, and 83 women were assigned to the control group. The average maternal age was around 32 years. Overcoming COVID-19 or vaccination against COVID-19 had no effect on the gestational age, weight, or height of the newborn. In all three groups of women, the most common mode of delivery was vaginal delivery, regardless of the recovery from COVID-19 or being vaccinated. There were no differences between groups in gravida and para parameters. Analysis of oxidative damage markers of the oxidative stress showed no significant difference between groups (*p* = ns) (Figure 2). The average concentration of ecDNA in the control group was 9.29 ± 6.63 ng/mL. In the COVID-19-recovered and vaccinated-against-COVID-19 groups, the average ecDNA concentrations were 10.40 ± 8.94 ng/mL and 11.32 ± 5.99 ng/mL, respectively, and no differences between groups were confirmed (H = 2.65, *p* = 0.26). No differences were observed regarding the DNase activity (F = 0.756, *p* = 0.47) (Figure 3). The average DNase activity in the control group was 5.8 ± 2.3 KU/mL. In women recovered from COVID-19, the average DNase activity was 5.08 ± 1.74 KU/mL and in the group of women vaccinated against COVID-19 it was 5.9 ± 2.88 KU/mL (Figure 3).

## 4. Discussion

Our results show that infection with SARS-CoV-2 and vaccination against COVID-19 did not cause any major effects on the clinical outcomes of pregnancy. Similarly, no differences were found in markers of oxidative damage to lipids or proteins between the groups. The lack of differences was supported by similar concentrations of ecDNA and DNase activity across groups.

Previous studies described that SARS-CoV-2 infection during pregnancy could lead to damage to placenta tissues because of oxidative imbalance [24,34,35]. Formed oxidative stress can thus interfere within the cell and damage the structures of the cells as lipids, proteins, and, in the last stage, nucleic acids [36]. Lipids are the first degree in which oxidative disbalance can influence macromolecules [37]. The representative marker of lipid peroxidation is TBARS [26]. Higher TBARS were reported in the plasma of women diagnosed with COVID-19 compared with controls [38]. These results are in contrast with our results. In our study, placental TBARS did not reveal differences between the study groups. A possible explanation for the discrepancy could be the different timing of infection. While Rolfo et al. focused on women positive for SARS-CoV-2 during the third trimester, in our study, women were infected during different time points of the pregnancy, primarily in the second trimester. It was published that maternal infection does not always lead to placental infection or to intrauterine transmission [39]. Explanation of the inconsistent results between our and Rolfo’s study could be in the short-term effects of SARS-CoV-2 on the placenta. Time might be an important confounding fact in the immune consequences of the infection [40]. In our study, we were also focused on the consequences of vaccination against COVID-19. Women were vaccinated in the third trimester and the average time difference between the vaccination and the placenta collection was 5 weeks. Our results are in line with previous studies showing that vaccination against COVID-19 does not lead to pathological changes in the placenta or clinical complications such as preterm birth [12,14]. This study shows no harmful effect on the selected markers of oxidative damage to lipids and proteins, nor on antioxidant capacity. These consequences are encouraged by the results of the quantification of ecDNA [41,42]. EcDNA is, in physiological conditions, rapidly removed by the DNases. During infections, autoimmune diseases, or tissue damage, the concentration of ecDNA can be elevated. Behind the higher ecDNA concentration can stand processes such as immune reactions, more frequent cell death, and insufficient ecDNA cleavage [43,44]. Increased concentrations of plasma ecDNA are associated with inflammatory processes and also in pregnancy complications such as preeclampsia and preterm birth [45,46]. In our study, clinical data showed no differences in the case of the preterm birth. These results agree with the outcomes of the concentration of ecDNA, as no difference was found in cord blood plasma ecDNA between the groups in our study. Previously, it has been published that the metabolism of mtDNA is affected by SARS-CoV-2 infection. In women infected during the third trimester, higher DNA oxidative damage was shown. However, these results correlated negatively with the concentration of mtDNA [47]. Mandó explained reduced mtDNA with the suppression of respiratory activity [47,48,49]. In our study, we did not observe any differences in the markers of oxidative stress damage. We also expected higher mtDNA with pro-inflammatory properties of mtDNA mediated by Toll-like receptor 9 [50]. However, similarly to total ecDNA, mtDNA and ncDNA were comparable between the groups, suggesting that neither SARS-CoV-2 infection nor vaccination affects cord blood plasma ecDNA of any subcellular origin.

From the clinical perspective, there were no major differences between the groups of participating women. Neither SARS-CoV-2 nor vaccination against COVID-19 had any impact on the outcomes, including preterm birth, birth weight and height, or the Apgar score. One of the limitations of the study is the lack of structural and functional analysis of the placenta. It was reported that infection with SARS-CoV-2 can interfere with the microbiota [17,18]; however, this analysis was not performed either.

A major limitation of this study is the small cohort of women with SARS-CoV-2 infection or vaccination during pregnancy. This is mainly related to the fact that the study was conducted in one hospital in a short period of time. In Slovakia, at that time, vaccination against COVID-19 during pregnancy was rarely performed. In addition, there was low confidence of the population in vaccination. Closely associated limitations of this study are the missing clinical data about the severity of the symptoms of the infection with SARS-CoV-2 and the vaccination against COVID-19. We admit that these can vary and affect our results. Unfortunately, these clinical data about the women participating in the study are not available. Another limitation is the lack of monitoring of the environment and profession of the women, as these can influence markers of oxidative stress. It would also be interesting to analyze ecDNA or oxidative stress in the plasma from the mothers. This analysis will be included in the follow-up study.

Despite negative results, the present study contributes to the literature on placental effects of COVID-19, which is likely affected by publication bias. With a wide palette of biomarkers of oxidative damage and antioxidant status, albeit on a small cohort, we have shown that oxidative stress is unlikely to be a common consequence of COVID-19 on the placenta. Similarly, no effect or even a trend was seen in placenta samples from women vaccinated against COVID-19 during pregnancy. To our knowledge, this is the first study that analyzed the concentration of ecDNA and DNase activity in umbilical cord blood plasma after exposure to SARS-CoV-2 during pregnancy. To conclude, our results indicate that infection with SARS-CoV-2 and vaccination against COVID-19 have no adverse effect on placental redox homeostasis or ecDNA in umbilical cord plasma.

## Figures and Tables

**Table 1:** Clinical characteristics of participants. Values are presented as mean ± standard deviation. (100-CS—vaginal delivery, CS—Cesarean delivery, B—before pregnancy, 1—first trimester, 2—second trimester, 3—third trimester, ns—non-significant).

Parameter	Controls	Recovered from COVID-19	Vaccinated against COVID-19	*p*-Value
Number of subjects COVID-19 recovered/vaccinated B/1/2/3 (n)	n = 83	n = 23 6/7/9/1	n = 20 0/0/0/20	
Maternal age (years) Cardiovascular diseases (n) Endocrine diseases (n) Neurological diseases (n) Psychiatric diseases (n) Gynecological disorders (n) Autoimmune diseases (n) Gastrointestinal diseases (n) Infections (n) Allergies (n) Smoking (n) BMI	33.2 ± 4.3 7 8 1 1 8 6 4 1 26 2 23.3 ± 3.9	31.0 ± 4.5 2 2 1 - - 1 - - 8 1 23.5 ± 5.1	33.1 ± 5.08 1 2 - - - 1 1 - 6 - 21.3 ± 3.3	ns ns ns ns ns ns ns ns ns ns ns ns
Previous miscarriage (n)	18	2	3	ns
Preterm birth < 37 week	2	1	-	ns
Birth weight (g)	3448 ± 531.9	3435 ± 475.3	3421 ± 411.9	ns
Birth height (cm)	50.5 ± 2.2	50.8 ± 1.7	50.4 ± 1.8	ns
GA (weeks)	40.0 ± 1.2	40.0 ± 1.5	40.1 ± 1.1	ns
Apgar score at 1 min	9.5 ± 1.1	9.3 ± 1.3	9.2 ± 1.5	ns
Apgar score at 5 min	9.8 ± 0.6	9.8 ± 0.9	10.0 ± 0	ns
Apgar score at 10 min	10.0 ± 0.2	10.0 ± 0.20	10 ± 0	ns
Gravida	1.9 ± 1.1	1.7 ± 0.8	2.1 ± 1.1	ns
Para	0.6 ± 0.8	0.5 ± 0.6	0.9 ± 0.8	ns
Mode of delivery (100-CS) %	97.6	95.7	90	
Mode of delivery (CS) %	2.4	4.3	10	
COVID-19 recovered/vaccinated B/1/2/3		6/7/9/1	0/0/0/20	

## Data Availability

All of the data is contained within the article.
